# Gluten hydrolyzing activity of *Bacillus* spp isolated from sourdough

**DOI:** 10.1186/s12934-020-01388-z

**Published:** 2020-06-12

**Authors:** Bennur Somashekharaiah Rashmi, Devaraja Gayathri, Mahanthesh Vasudha, Chakra Siddappa Prashantkumar, Chidanandamurthy Thippeswamy Swamy, Kumar S. Sunil, Palegar Krishnappa Somaraja, Patil Prakash

**Affiliations:** 1 Department of Microbiology, Davangere University, Davangere, Karnataka 577002 India; 2 SDM Research Institute for Biomedical Sciences (SDMRIBS), Shri Dharmasthala Manjunatheshwara University, Manjushree Nagar, Dharwad, Karnataka 580 009 India; 3 Present Address: Central Research Laboratory, K S Hegde Medical Academy, Deralakatte, Mangaluru, 575018 India

**Keywords:** *Bacillus*, Celiac disease, Gliadin, Gluten, Mass spectrometry

## Abstract

**Background:**

Celiac disease is an intestinal chronic disorder with multifactorial etiology resulting in small intestinal mucosal injuries and malabsorption. In genetically predisposed individuals with HLA DQ2/DQ8 molecules, the gluten domains rich in glutamine and proline present gluten domains to gluten reactive CD4^+^ T cells causing injury to the intestine. In the present experimental design, the indigenous bacteria from wheat samples were studied for their gluten hydrolyzing functionality.

**Results:**

Proteolytic activity of *Bacillus spp*. was confirmed spectrophotometrically and studied extensively on gliadin-derived synthetic enzymatic substrates, natural gliadin mixture, and synthetic highly immunogenic 33-mer peptide. The degradation of 33-mer peptide and the cleavage specificities of the selected isolates were analyzed by tandem mass spectrometry. The gluten content of the sourdough fermented by the chosen bacterial isolates was determined by R5 antibody based competitive ELISA. All the tested isolates efficiently hydrolyzed Z-YPQ-pNA, Z-QQP-pNA, Z-PPF-pNA, and Z-PFP-pNA and also cleaved 33-mer immunogenic peptide extensively. The gluten content of wheat sourdough was found to be below 110 mg/kg.

**Conclusion:**

It has been inferred that four *Bacillus spp* especially GS 188 could be useful in developing gluten-reduced wheat food product for celiac disease prone individuals.

## Background

Celiac disease (CD) is a multifactorial disorder of the small intestine in genetically susceptible individuals caused by an inappropriate immune response to ingested wheat gluten and similar proteins of barley and rye [1]. The pathogenesis of disease involves interactions amongst environmental, genetic and immunological factors [2]. CD is one of the HLA linked disorders and the genes encoding for HLA-DQ molecules namely DQ (α1*0501, β1*02)/DQ2 or to a lesser extent DQ (α1*03, β1*0302)/DQ8 which are present on antigen presenting cells, are considered as principal genetic factors responsible for CD. These HLA molecules preferentially present tissue transglutaminase (tTG) deamidated proline- and glutamine-rich peptides to gluten reactive CD4^+^ T cells causing intestinal damage, which can be characterized by partial and subtotal villous atrophy with hyperplastic crypts, increased intra epithelial lymphocytes and mononuclear infiltration in the lamina propria [3–5]. Earlier workers have reported that CD was limited to the Western population due to their gluten-rich diet [6, 7], but nowadays the prevalence of CD is reported to be worldwide. The prevalence of CD has increased significantly over the past two decades, especially around 1 in 100 of the general population with female to male ratio ranging from 1:3 to 1.5:1 in Western countries as per World Gastroenterology Organization data [8]. Reports on serological diagnosis of CD in developing countries in the Middle East, North Africa and Asia have however revealed that the prevalence of CD in these regions compares well to the Western population [9, 10]. The prevalence of CD in low-risk population expressing lower risk genetic factors like HLA DQ 2.2 is 0.14–5.7% while 1.2–55% in high-risk population [11] has been adequately recorded in the developing world. The worldwide increased consumption of gluten-rich foods and predisposing genotypes might be attributed to the widespread and universal emergence of CD.

Several non-dietary therapies such as blockage of tissue transglutaminase activity, oral administration of permeability inhibitors like zonulin, use of polymers that sequester ingested gliadin peptides and corticosteroids have been suggested to treat CD [12–15]. However, safety, efficacy and monitoring of long-term treatment effect of these non-dietary therapies are questionable. Strict adherence of life-time exclusion of gluten from the diet has proven to improve the clinical symptoms of CD and was found to be safe without side effects and therefore, gluten-free food remains the mainstay for CD management [16]. Gluten is rich in proline and glutamine residues and undergoes partial digestion by gastric, pancreatic and intestinal brush border enzymes, making gluten degrading microbial enzymes in the higher hierarchy of choice in developing gluten-free foods. Several studies reported the use of microorganisms from human and other origins for gluten hydrolysis [16, 17] and in our earlier study, the probiotic attributes of the gluten hydrolyzing bacteria [18, 19] has been reported. In the present study indigenous strains of *Bacillus* spp (accession no: GS1KX272351, GS 181 KX272352, GS 188 KX272353, GS 547 KX272354, GS 33 KX272356, GS 3 KX272355, GS 143 KX272357 and GS 199 KX272358) isolated from wheat samples with probiotic potential were characterized for their gluten hydrolyzing functionality. The ability of these isolates to target the celiac epitopes particularly 33-mer peptide from gliadin were studied using tandem mass spectrometry. Further, the reduced gluten content in wheat sourdough fermented by the selected bacterial isolates, was determined using R5 antibody based competitive ELISA.

## Results

**Fig. 1:**
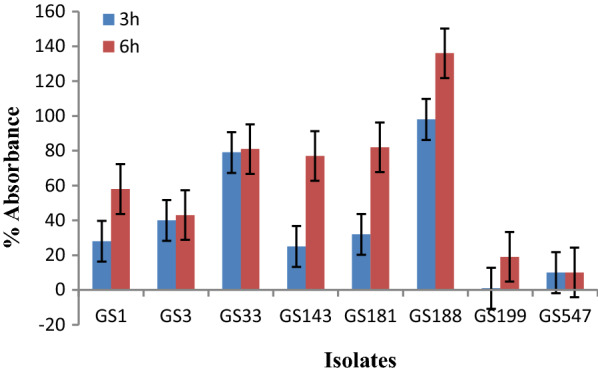
Proteolytic activity of bacteria isolated from wheat sourdough on gluten. Line graph was plotted incorporating sample mean (n = 3) and error bar (standard deviation) of individual isolate. The results were statistically significant at p < 0.05 when means of each treatment compared in pair to the means of other treatment at different time of incubation in Tukey’s HSD test in conjunction with ANOVA

Eight bacterial isolates (*Bacillus subtilis* GS 1 KX272351, *B. subtilis* GS 181 KX272352, *B. subtilis* GS 188 KX272353, *B. subtilis* GS 547 KX272354, *B. cereus* GS 3 KX272355, *B. subtilis* GS 33 KX272356, *B. cereus* GS 143 KX272357, and *B. cereus* GS 199 KX272358) showing gluten hydrolyzing function (Fig. 1) and the amino acids (lysine) released due to exoproteolytic activity of these isolates were listed in Table 1. However, GS 199 and GS 547 showed relatively low gluten hydrolyzing potential.

**Table 1:** Exoproteolytic activity mediated release of free amino acids

Isolates	Mg lysine/g of gluten mean ± SD*	Isolates	Mg lysine/g of gluten mean ± SD*
GS 1	10 ± 1.2	GS 181	15 ± 1.12
GS 3	12 ± 1.1	GS 188	17 ± 1.2
GS 33	11 ± 0.98	GS 199	20 ± 1.0
GS 143	12 ± 1.3	GS 547	20 ± 0.97

*The p-value corresponding to the F-statistic of one-way ANOVA was lower than 0.05, suggesting that the observations were significantly different. Tukey’s HSD post hoc analysis test was applied to each of the 28 pairs (where k = 8), and found the values statistically significant at p < 0.05

**Fig. 2:**
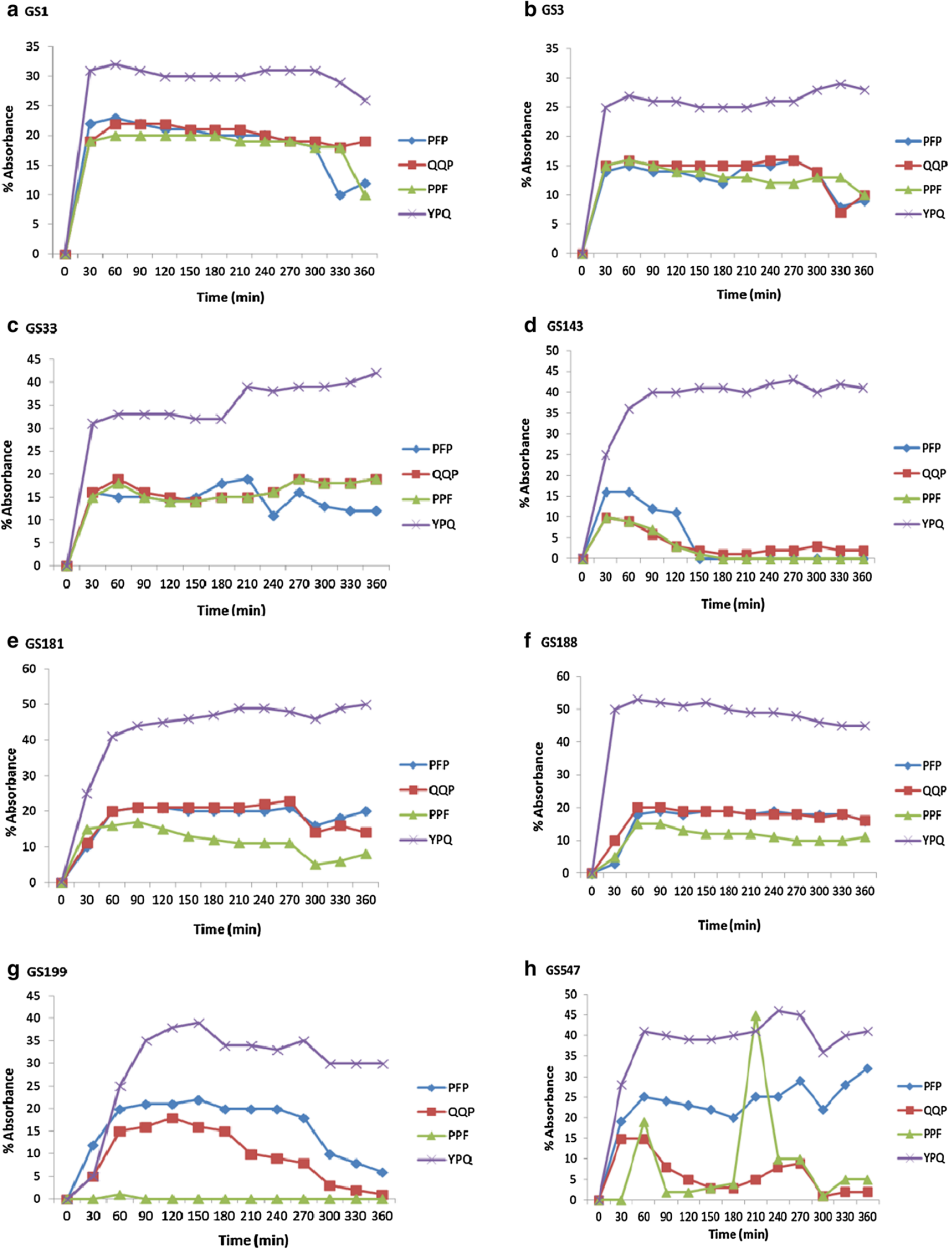
**a–h** Hydrolysis of gliadin derived enzyme substrates Z-YPQ-pNA, Z-PPF-pNA, Z-QQP-pNA and Z-PFP-pNA by **a** GS1; **b**GS 3; **c** GS 33; **d** GS 143; **e** GS 181; **f** GS 188; **g** GS 199; h GS 547. Line graph was plotted incorporating sample mean (n = 3) and error bar (standard deviation) of individual isolate. The results were statistically significant at p < 0.05 when means of each treatment compared to the means of other treatment pair wise in Tukey’s HSD test in conjunction with ANOVA

**Fig. 3:**
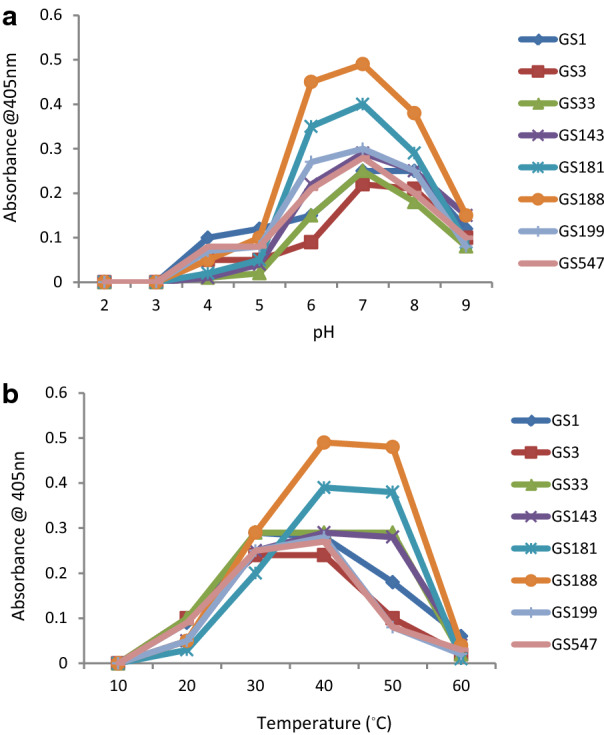
**a** Determination of Z-YPQ-pNA hydrolysis at different pH. Line graph was plotted incorporating sample mean (n = 3) and error bar (standard deviation) of individual isolate. In Tukey’s test followed by ANOVA, means of every pH treatment were compared to the means of every other treatment, the results were statistically significant at p < 0.05 except for the compared pair of means pH 2 and 3. **b** Determination of Z-YPQ-pNA hydrolysis at different temperature. Line graph was plotted incorporating sample mean (n = 3) and error bar (standard deviation) of individual isolate. The pair wise comparison of means of every temperature treatment were statistically significant at p < 0.05 when Tukey’s HSD test followed by ANOVA was applied to each of the treatment

The selected eight isolates were further subjected for the evaluation of enzymatic cleavage specificities using synthetic gliadin epitope derived tripeptide analogs namely: Z-YPQ-pNA, Z-QQP-pNA, Z-PPF-pNA and Z-PFP-pNA in which amino terminal was modified with benzyloxycarbonyl (Z) and served as protective group whereas carboxy terminal with *p*-nitro anilide (pNA) served as reporter group. Among the four substrates, Z-YPQ-pNA was most efficiently cleaved by all the 8 isolates although, the substrates such as, Z-QQP-pNA, Z-PPF-pNA and Z-PFP-pNA were also cleaved with variation (Fig. 2). In case of GS 199, Z-YPQ-pNA was cleaved to the maximum while Z-PPF-pNA was cleaved minimum. GS 547 also cleaved Z-YPQ-pNA to the maximum and interestingly Z-PPF-pNA was cleaved efficiently at 220 min although at other time internals the hydrolysis was low. There was no significant hydrolysis where the substrates incubated only with buffer. To further examine the effect of pH range over gliadin derived tripeptide cleavage by the tested isolates, Z-YPQ-pNA substrate hydrolysis was measured in solutions ranging in pH from 2.0 to 9.0. All the isolates showed maximum hydrolysis of Z-YPQ-pNA at pH 7 (Fig. 3a). Notably, substrate hydrolysis rates was less with low pH and at pH 2 or 3, none of the isolates hydrolyzed Z-YPQ-pNA.

The effect of temperature on Gliadin tripeptide Z-YPQ-pNA hydrolysis showed varied results. In which, GS 181, GS 188 exhibited highest hydrolysis between 40 and 50 °C, and GS 33, GS143 was between 30 and 50 °C, and the other four isolates (GS 1, GS 3, GS 199, GS 547) showed maximum hydrolysis between 30 and 40 °C. Further, as the temperature range decreased below 30 °C, the rates of hydrolysis were also decreased and at 10 and 60 °C, there was no hydrolysis detected (Fig. 3b).

**Fig. 4:**
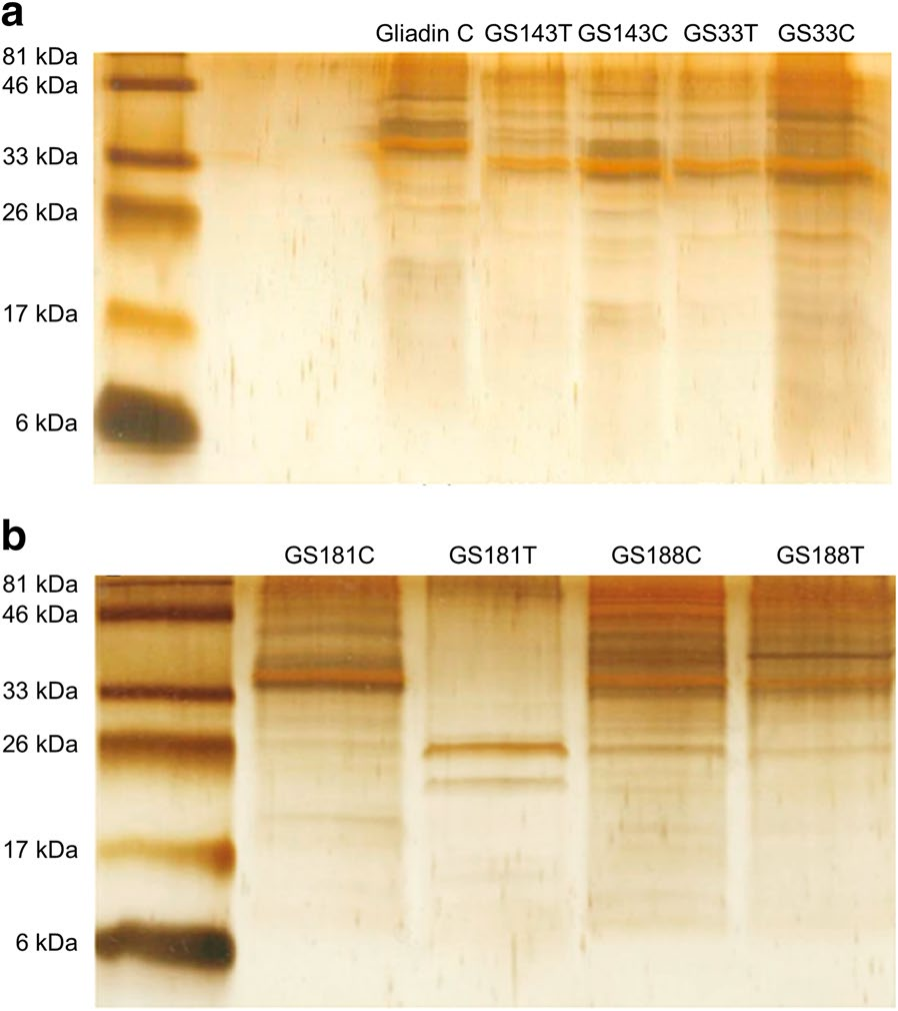
SDS-PAGE; Degradation of gliadin **a** by GS 33 and GS 143 and **b** by GS 181 and GS 188, control **c** at 0 h and test sample (T) after 6 h incubation

Four out of eight isolates viz., GS 33, GS 143, GS 181 and GS 188 showing high rate of gluten hydrolysis were chosen for further study. The degradation of gliadin by tested bacteria was investigated by incubating gliadin in the suspensions of GS 33, GS 143, GS 181 and GS 188. The aliquots of incubated samples were analyzed by SDS-PAGE. The clearance of protein bands in the bacteria treated samples compared to the control indicated the degradation of gliadin. After 6 h of incubation for all the four isolates with gliadin, the gliadin protein bands with molecular weight 81 kDa to 46 kDa had undergone substantial degradation while the gliadin proteins < 46 kDa were degraded markedly (Fig. 4a, b).

**Fig. 5:**
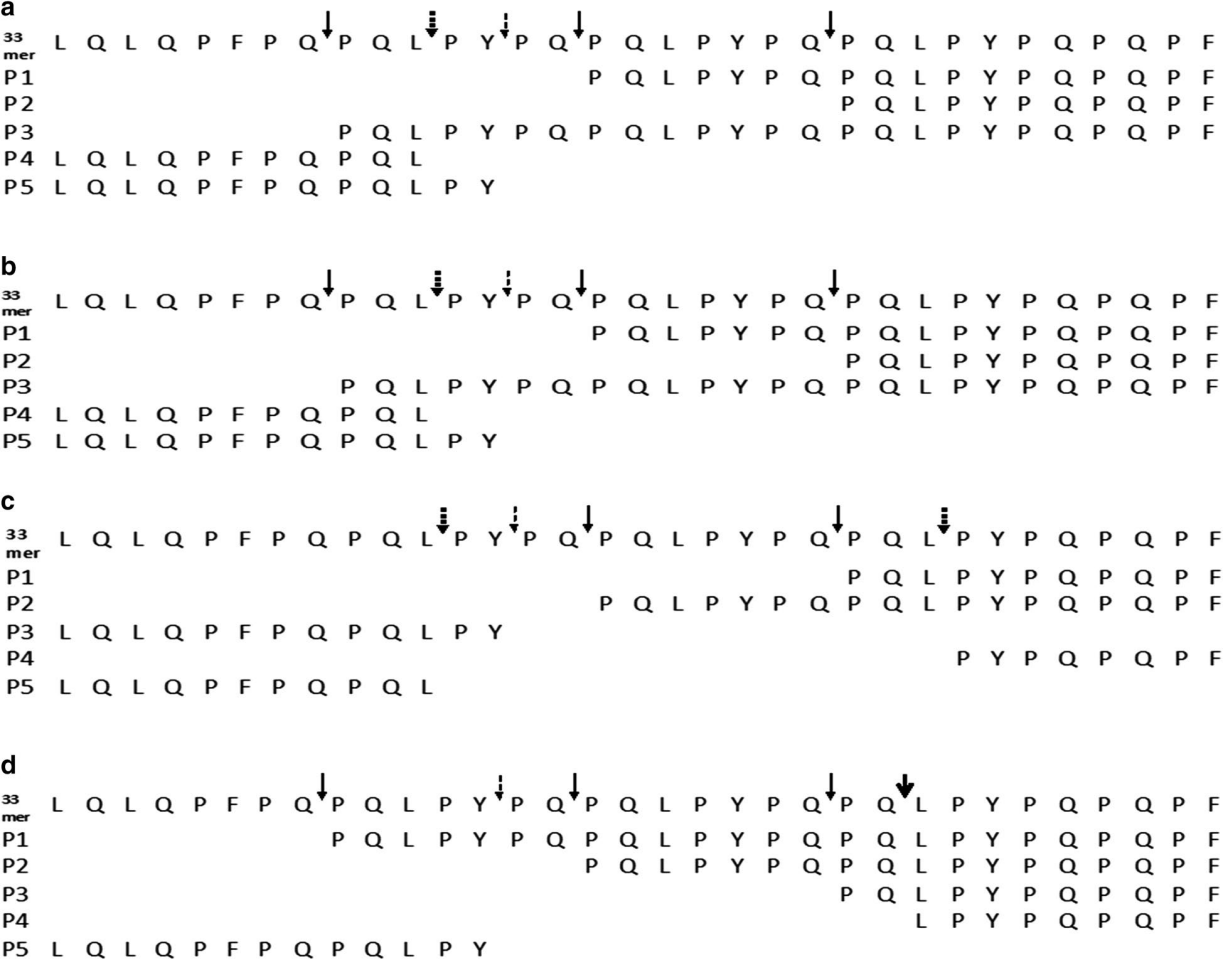
**a** Fragment cascade of 33-mer peptide degraded by GS 33, **b** Fragment cascade of 33-mer peptide degraded by GS 143, **c** Fragment cascade of 33-mer peptide degraded by GS 181, **d** Fragment cascade of 33-mer peptide degraded by GS 188

The 33-mer celiac super antigen derived from gliadin contained three immunogenic epitopes namely: DQ2-α-I, α9 (PFPQPQLPY) and DQ2-α-II, α2 (PQPQLPYPQ) and DQ2-α-III (PYPQPELPY). Therefore, the susceptibility of 33-mer gliadin peptide to proteolytic breakdown by four selected bacterial isolates was investigated by tandem mass spectrometric analysis (Additional file 1: Figure S1). The retention time for the intact 33-mer peptide was found to be 24.08 min. After 6 h of incubation, the degradation of 33-mer was evidenced by the appearance of degraded fragments eluting majorly between 14 and 24 min in all the tested isolates including the consortium and were indicated using arrows (Additional file 1: Figures S2a, b and Additional file 1: Figure S3). The LC MS/MS analysis of the fragmentation cascade of the 33 mer peptide after incubation with tested bacterial suspensions were determined by comparing the raw MS data to the database sequence for gliadin protein identification on ProteinLynx Global Server (PLGS) and the matched peptides with high scores were considered (Fig. 5 and Additional file 1: Figure S4). Further, the enzymatic cleavage site specificities of the four investigated bacteria could be derived from the N and C termini of the degraded fragments. In all the four isolates and their consortium, cleavage was observed between Q ↓ P, L ↓ P, Y ↓ P amino acid residues of the 33-mer. Interestingly, GS 188 cleaved between Q ↓ L also. However, on Z-YPQ-pNA hydrolysis, prominent cleavage was noticed after YPQ↓ consistently. Most of the degraded fragments were the resultant of cleavage at YPQ↓, LPY↓, and PQL↓ with site specific cleavage at FPQ↓ and/or at other positions (Additional file 1: Figure S5).

Further, in the present study, microbial growth and proteolysis during sourdough fermentations using the four tested bacterial isolates namely: GS 33, GS 143, GS 181, GS 188 and their consortium were determined. Aseptic doughs at pH 3.5 and 6.5 were used as acidic and neutral controls respectively to know the proteolysis and gluten macro polymers modification in the absence of bacterial enzyme activity. The cell counts were listed in Table 2, pH and amino nitrogen contents were shown in Additional file 1: Figure S6a, b. In neutral sourdough at 0 and 6 h of incubation no growth of bacteria was observed, and after 24 and 48 h 10^4^/CFU was observed, while no growth was observed in CAD.

**Table 2:** Cell counts of sourdough samples

Fermentation time (h)	Neutral control	CAD	GS 33	GS 143	GS 181	GS 188	Consortium
0 h							
Cell counts cfu/g*	–	–	2X10^5^ ± 0.1798	6X10^5^ ±0.0197	5X10^5^ ± 0.0244	7 X10^5^ ± 0.0258	5X10^5^ ±0.0336
6 h							
Cell counts cfu/g*	–	–	6X10^6^ ± 0.1317	3X10^7^ ± 0.216	5X10^6^ ±0.149	4X10^7^ ±0.173	8X10^6^ ±0.145
24 h							
Cell counts cfu/g*	> 10^4^	–	7X10^8^ ±0.1348	1X10^9^ ± 0.1536	5X10^8^ ±0.247	1X10^9^ ±0.1744	8X10^8^ ±0.1846
48 h							
Cell counts cfu/g*	>10^4^	–	1X10^9^ ±0.1978	2X10^9^ ±0.238	1X10^9^ ±0.318	2X10^9^ ±0.427	1X10^9^ ±0.854

*cell counts were expressed as mean ± standard deviation and the values were statistically significant at p<0.05

RIDASCREEN^®^ Gliadin competitive ELISA was used to determine the amount of gluten reduced during the fermentation of wheat sourdough by tested bacteria. The absorption was considered inversely proportional to the gliadin with respect to prolamin concentration in the sample (Table 3). The results were calculated using cubic spline function in RIDA^®^SOFT Win software. The gliadin concentration in ng/ml was read from the standard curve and further multiplied by the dilution factor of 500. The results were expressed in mg gliadin/kg of food. For further calculation into gluten, a factor of 2 was used and the results were showed in Table 3.

**Table 3:** The amount of gluten at different intervals of fermentation

Isolates	0 h	6 h	24 h	48 h
GS 33	> 270.00	> 270.00	254.17	104.89
GS 143	> 270.00	> 270.00	> 270.00	111.62
GS 181	> 270.00	> 270.00	> 270.00	107.18
GS 188	> 270.00	> 270.00	267.59	98.86
Consortium	> 270.00	> 270.00	263.56	102.10
CAD	> 270.00	239.39	13.05	< 10.00
Neutral dough	> 270.00	> 270.00	> 270.00	> 270.00

These results were calculated using cubic spline function in RIDA®SOFT Win software. Gliadin/gluten concentration was determined using standard curve and multiplied by dilution factor of 500

## Discussion

Immunogenic gluten peptides involved in celiac disease (CD) are rich in proline and glutamine residues and are resistant to human digestive enzymes. The lack of post prolyl activity of human digestive enzymes result in partial digestion of ingested gluten as trypsin needs arginine or lysine residue at P1 position, whereas pepsin requires phenylalanine, tyrosine, tryptophan or leucine at P1 position of the binding peptide while phenylalanine, tyrosine or tryptophan at P1 are of preference for chymotrypsin activity [20]. However, several microbial proteases are reported with significant gluten hydrolyzing activities. Noticeably, *Lactobacillus sanfranciscensis, L. alimentarius, L. brevis, L. hilgardii, Myxococcus Xanthus, Sphyngomonas capsulate, Flavobacterium meningosepticum, Rothia spp., Aspergillus niger* were reported for their gluten hydrolyzing activities [21–24].

Gluten hydrolysis by selected bacterial isolates in the present study, showed that the tripeptide YPQ, QQP, PPF, PFP occur frequently in gliadin. The observed cleavage specificities at XPQ↓, XFP↓, XQP↓ and XPF↓ tripeptides indicated that multiple epitopes in gliadins would be targeted, credibly destroying their gliadin specific T cell inducing activities. YPQ is one of the relatively abundant tripeptides particularly in 33-mer peptide (LQLQPFPQPQLPYPQPQLPYPQPQLPYPQPQPF) of a2-gliadin which has been considered as celiac super antigen and used as a model epitope in many in vitro studies concerning celiac disease and gluten hydrolysis, whereas, QQP and PFP occur frequently in 26-mer peptide (FLQPQQPFPQQPQQPYPQQPQQPFPQ) of γ-gliadin- another predominant celiac epitope [25, 26]. However, *Rothia mucilaginosa* and *R. aeria* rapidly cleaved YPQ whereas QQP, PFP and PPF tripeptides were not hydrolyzed, while anaerobic *Bifidobacteria spp.* were unable to cleave none of these tripeptide substrates [27].

SC PEP, AN PEP, EP-B2 are the major glutanase enzymes and their application regarding gluten intolerance [23, 28, 29] have been reported. These enzymes exhibited unique cleavage at XPQ↓, while in an another study *Rothia* enzymes cleaved at XPQ↓ as well as LPY↓ [27]. However, in the present study, the cleavage specificities of the tested isolates in 33-mer peptide were XPQ↓, LPY↓ and PQL↓. These multiple cleavage specificities observed in the present study resulted in extensive cleavage of 33 mer epitope, the specific 9- mer cores of the epitopes that binds HLA DQ2/DQ 8 binding motifs. Additionally, deamidation of glutamine residues in 9-mer cores at specific positions renders the epitopes to bind HLA DQ2/DQ8 with high affinity [30, 31]. Particularly, QLP motif has been identified as one of the major tTG recognition sequences in the gliadin peptides [32]. Interestingly, the cleavages observed in the present study were between Q↓P, L↓P, Y↓P, and Q↓L residues in the 33-mer peptide. In that, the cleavage between Q↓L and L↓P will target QLP motif, probably reducing specificity of the epitope towards tTG binding.

The effect of pH on tripeptide hydrolysis on all the selected isolates showed maximum hydrolysis at pH 7, these findings were in agreement with the study where the optimal activities of *R. aeria* and diminished in parallel with decreasing pH [27]. These in vitro results showed that the gliadin degrading enzymes of tested *Bacillus spp.* were not inactivated at pH 4 to 5 suggesting that during fermentation process as the pH decreases, the gluten degrading activity would still be retained.

At 6 h of fermentation, the pH was 4.5 to 5.5 with exponentially growing cells, however at 24 h the pH was 3.6 to 3.8 and resulted in the cessation of growth and proteolytic activity in the dough [33]. Whereas, in the present study after 48 h, the pH was between 4.5 and 4.7 with an increased cell counts suggesting that slow decrease in the pH facilitated the growth and metabolic activity of bacteria throughout the fermentation process. The proteolysis in doughs was determined as a measure of amino nitrogen content [34]. In this study, the amino nitrogen content has increased almost four times at the end of the fermentation indicating the significant proteolysis in the dough.

Usage of gluten-free or reduced food is the only available strategy for the management of CD. In view of this, sourdough fermentation can be considered as an effective method to reduce the immunogenicity of the wheat foods without the complete removal of gluten. Further, several studies demonstrated the possibility of sourdough fermentation as a measure to reduce the level of gluten in wheat foods [5, 21]. Furthermore, complete degradation of gluten requires long fermentations with a combination of several strains of starter culture and/or with potential peptidases [5, 21, 35]. According to the Codex Alimentarius, two categories for labeling of food according to the gluten content: (a) Food products which contain less than 20 mg/kg (ppm) can be labeled as “gluten-free”. (b) Food products labeled as “very low gluten” can have a gluten content above 20 and up to 100 mg/kg (ppm) [36]. Accordingly, in the present study the gluten content of the wheat sourdoughs fermented by four tested *Bacillus spp.* and their consortium was evaluated by the R5 antibody based competitive ELISA. In R5 based ELISA, no significant reduction in the gluten content was observed for all the four tested isolates including the consortium at 6 and 24 h of fermentation (Table 3). However, at 24 h of fermentation, GS 33, GS 188 and the consortium of the four isolates reduced gluten content to 254.17 mg/kg, 267.59 mg/kg, and 263.56 mg/kg respectively. After 48 h of fermentation, the gluten content reduced by the four isolates ranged between 98 and 112 mg/kg. These findings suggested that the sourdough fermented by these isolates perhaps can be used in the preparation of foods for celiac disease prone individuals.

In CD condition, human digestive enzymes are unable to reduce gluten immunogenicity, the four tested *Bacillus spp.* indigenous to wheat had shown promising gluten hydrolysis. Present study revealed that the multiple epitopes in gliadins would be targeted by the tested isolates, credibly destroying their gliadin specific T cell inducing activities. Therefore, the indigenous *Bacillus* spp. chosen in the present study are competitive, safe and promising gluten hydrolyzing candidates. Further, demonstration of safety and efficacy in vivo would certainly be useful either alone or in combination promote the development of probiotic treated gluten reduced wheat foods for people with celiac disease.

## Materials and methods

### Bacterial isolates

In our earlier study, 260 gluten hydrolyzing bacteria were isolated, out of which only eight most promising isolates were identified by 16S rDNA typing and also characterized for probiotic potentiality. The eight isolates, were identified by 16S rDNA typing (*Bacillus subtilis* GS 1 KX272351, *B. subtilis* GS 181 KX272352, *B. subtilis* GS 188 KX272353, *B. subtilis* GS 547 KX272354, *B. cereus* GS 3 KX272355, *B. subtilis* GS 33 KX272356, *B. cereus* GS 143 KX272357, and *B. cereus* GS 199 KX272358) [18] and these eight isolates were used in the present study.

### Qualitative screening on gluten agar plates

Qualitative analysis of gluten hydrolysis by sourdough isolates was performed using gluten agar plates. Upon solidification of autoclaved gluten agar [37], 6 mm wells were punched using sterile cork borer and inoculated with 100 μl (OD 1.2 at A_610_) of 18 h old bacterial culture (LB) and incubated at 37 °C for 48 h. After incubation, the agar plates were stained using Coomassie Blue for 30 min (Coomassie Brilliant Blue G-250;1 g, methanol;400 ml, glacial acetic acid;100 ml, distilled water; 500 ml) and subsequently destained overnight using destaining solution. Then the clear zone around the well indicating gluten hydrolysis, was measured. The experiment was performed in triplicates.

### Spectrophotometric assay of gluten hydrolysis

The selected bacterial isolates with clear zone of gluten hydrolysis on agar plates above the mean value 10 mm (including well diameter) were selected further for quantitative analysis of gluten hydrolysis spectrophotometrically. During the quantification assay, increased absorption in trichloro acetic acid solution with time was measured as proteolytic breakdown of gluten [37, 38]. In brief, selected bacteria were cultured in Luria–Bertani broth (LB) for 24 h and centrifuged at 503 xg/15 min (Microspin, Eppendorf, USA). The pellets were washed thrice using 1 M Tris–HCl buffer (pH 7) and OD was adjusted to 1.2 at 610 nm. Further, 25 ml of solution I (Tris–HCl (mol/l);0.5%, (0.2%) sodium azide;4%, CaCl_2_.2H_2_O;0.074%, and gluten;0.8%)) was inoculated with 1 ml of bacterial suspension and incubated at 37º C in shaking water bath for 6 h. At regular intervals 0 h, 3 h and 6 h, the solution was sampled by adding 1 ml of stopping reagent (Trichloracetic acid:1.634%/sodium acetate:1.804%/acetic acid:1.886% was added to arrest the enzyme activity) and kept at room temperature for 30 min. Then the samples were centrifuged at 10,956x*g*/10 min (Microspin, Eppendorf, USA) and the absorption of supernatants was measured at 275 nm using quartz cuvette of path length 1 cm spectrophotometrically (Thermo Scientific^TM^ ND 2000c, USA). Each sample was measured in triplicates. Solution I containing 1 M Tris–HCl alone (without bacterial cells) served as blank and its value was subtracted each time from the absorption values of samples. Further, the absorption values of cell suspension in solution I without gluten was used to tare the TCA soluble material released due to autolysis of cells. In addition, absorptions at time 0 h were subtracted from subsequent readings (3 h and 6 h).

### Colorimetric estimation of free amino acids

The amino group of free amino acids forms a complex with cadmium ninhydrin, so amino acids released due to exoproteolytic activity were colorimetrically measured using cadmium ninhydrin reagent [37]. For the assay, the bacterial cells were inoculated to LB incubated for 6 h as described in the spectrophotometric method. Further, 10 ml of the sample was centrifuged at 503x*g*/15 min (Microspin, Eppendorf, USA) and the supernatant was added to the cadmium-ninhydrin reagent (0.8 g cadmium-ninhydrin: 80 ml of absolute ethanol; 10 ml acetic acid (AR), and 1 g CaCl_2_/1 ml distilled water) in 1:2 ratio and further heated at 84 °C for 5 min. After cooling, the absorption was measured at 507 nm. The absorption of a blank containing ninhydrin reagent and 1 ml distilled water was subtracted and the experiment was performed in triplicates.

### Degradation of gliadin epitope derived *p*-nitroanilide substrates

The benzyloxycarbonyl-XXX-*p*-nitroanilide substrates (Z-YPQ-pNA, Z-QQP-pNA, Z-PPF-pNA and Z-PFP-pNA) were chemically synthesized were purchased from GenScript (USA). All these substrates were dissolved in 75% (v/v) dimethyl sulfoxide to make up to final concentration of 10 mM. Selected bacterial isolates were grown on gluten agar media for 48 h at 37 °C, then suspended in Tris–HCl (20 mmol/l) solution (pH 8.0), washed thrice and diluted to make up OD_620_ = 1.2. Further, 4 μl of each substrate was incubated separately with 200 μl of bacterial suspension in 96-well microtitre plate and the plates were sealed with optical adhesive covers (Applied Biosystems, USA). The hydrolysis of peptide was determined from the proteolytic removal of pNA group, which was monitored at 405 nm. Absorbance readings were taken at every 30 min interval from 0–6 h using a microtiter plate reader (Bio-Rad 680, USA). The reaction mixture containing heat killed bacteria with substrate was used as control. Further, optimum pH and temperature for gluten hydrolysis were determined by incubating the reaction mixtures at different pH (pH 2 to 9) and temperature (10 to 60 °C) ranges [39]. The experiment was performed in triplicates.

### Gliadin degradation in solution

Gliadin solution was prepared (cat no: 101778, MP bio medicals, France) by dissolving 5 mg/ml in 60% (v/v) ethanol. To which selected bacterial suspension (1.2 OD at 620 nm) was added to make up final concentration of 250 μg/ml and incubated (37 °C). At 0 and after 6 h of intervals, aliquots of 100 μl of incubation mixtures were collected; heat inactivated in boiling water bath for 10 min and vacuum dried. Further, the dried samples were resuspended in 1X SDS sample loading buffer and analyzed on SDS PAGE (12%) (constant voltage of 80 V/120 min) (GeNei, India). The gels were subjected to silver nitrate staining [40] and observed for protein bands.

### Degradation of 33-mer gliadin peptide in solution

The degradation of 33-mer peptide by sourdough isolated bacteria was performed as described previously with slight modifications [38, 39]. The 33-mer (LQLQPFPQPQLPYPQPQLPYPQPQLPYPQPQPF) gliadin derived peptide was chemically synthesized at a purity of 90% (Genscript, USA) and dissolved in milliQ water to make up to the concentration 10 mg/ml. Further, 33-mer solution was added to the bacterial suspension (1.2 OD at 620 nm) to the final concentration 250 μg/ml and incubated at 37 °C. After 6 h of incubation, 100 μl aliquot was collected and boiled to inactivate the enzyme activity, filtered using 0.22 μm membrane filters (SF13, HiMedia Laboratories, India), vacuum dried and subjected to tandem mass spectrometry.

### Liquid Chromatography tandem mass spectrometry using QTOF

For the characterization of 33-mer fragments the sample was dissolved in 20µL of 5% acetonitrile with 0.1% Formic acid. After centrifugation (10000x*g*) 10 µl was injected onto reversed phase C18 UPLC column (Acquity UPLC BEH C18 Column (P/N-186003556): 2.1 mm X 150 mm, 1.7 µm, 130A˚, pH Range – 1 to 12). Buffer A consisted of 0.1% formic acid in MS grade water and buffer B contained 0.1% formic acid in acetonitrile. Further, peptides were eluted using a linear gradient of buffer B from 0% to 80% over a 60 min time interval at a flow rate 0.3 µl/min. The peptides separated on UPLC were then directed to Q-TOF instrument (Waters Synapt G2) for Mass Spectrometric analysis with following experimental instrument parameters- polarity: ES + , analyser: resolution mode, capillary: 3.5000 kV, source temperature: 120 °C, sampling cone: 40.0000, extraction cone: 5.0000, desolvation temperature: 350 °C, cone gas flow: 30 L/Hr, and desolvation gas flow: 800 L/Hr. Then the raw MS/MS data obtained was processed by MassLynx 4.1 WATERS. The individual peptides MS/MS spectra were matched to the database sequence for protein identification on ProteinLynx Global Server (PLGS), WATERS. The search parameters in gliadin database included missed cleavage (1) with 50 ppm and 75 ppm peptide and fragment tolerances respectively.

### Wheat sourdough fermentation

The characteristics of the wheat flour (dry weight) used in the present study were as follows: 12.8% moisture content, 10.8% protein, 1.6% fat, 1.85% fibre, 1.75% minerals, 0.6% ash content, and 1.5% total soluble carbohydrate. Sourdough contained 80 g of wheat flour, 320 g of water and inoculum of final concentration 1X10^5^ CFU/g of dough. Aseptic dough with erythromycin (2 mg) served as neutral dough. Whereas aseptic dough adjusted to pH 3.5 using a mixture of lactic and acetic acids at a ratio 4:1 served as chemically acidified dough (CAD). Further, to determine the cell counts during fermentation, dough samples were collected at 0, 6, 24 and 48 h of fermentation, serially diluted, plated onto the nutrient agar medium and incubated at 37 °C for 24 h. To determine the dough pH, dough (1 g) was mixed with deionized water (9 ml) and vortexed for 5 min. Then using a glass electrode (Remi, India) pH was measured. The total amino nitrogen content was determined by the modified ninhydrin method [41]. Glycine standard curve was used to express the amino nitrogen content as millimoles of glycine per kilogram of dough. The experiment was performed in triplicates.

### R5 antibody based competitive ELISA

The gliadin content in fermented sourdough was determined using a R5 antibody based competitive ELISA kit (RIDASCREEN^®^ Gliadin competitive, R7021, Germany) according to manufacturer’s instructions. In brief, sample (1 ml) was mixed thoroughly with 60% ethanol (9 ml) and kept on a rotary shaker for 10 min. Then the sample was centrifuged for 10 min/1132x*g* at 25 °C and the supernatant was diluted with sample diluent buffer in 1:50 ratio. Thus diluted sample (50 μl) was added to gliadin coated microtiter wells and further to which equal volumes of enzyme conjugate was added, mixed gently and incubated at 25 °C for 30 min. The wells were washed thrice using wash buffer and then substrate/chromogen (100 μl) was added and mixed gently. After incubation at 25 °C for 10 min in dark, stopping reagent (100 μl) was added and mixed gently and absorbance was measured at 450 nm. The experiment was conducted in triplicates and the results were calculated using cubic spline function in RIDA^®^SOFT Win software available for RIDASCREEN immunoassays.

### Statistical analysis

All the experiments were conducted in triplicates and results expressed as mean ± standard deviation. Unless mentioned the data were analyzed using ANOVA and Tukey’s HSD post hoc test, the level of significance was set at p < 0.05 (GraphPad Prism version 5.02 for Windows, GraphPad Software, San Diego, California, USA).

## Conclusions

Celiac disease individuals shown to have small intestinal mucosal injuries and malabsorption of wheat based food, and thereby making gluten-free diet as mandatory for recommendation of celiac disease control. Indigenous bacteria from wheat samples may hold the key to ameliorate the discomfort caused to patients affected with celiac disease and were studied for their gluten hydrolyzing functionality. Out of eight tested isolates, four isolates efficiently hydrolyzed Z-YPQ-pNA, Z-QQP-pNA, Z-PPF-pNA, and Z-PFP-pNA and also cleaved 33-mer immunogenic peptide extensively. This study contributes to a better understanding of sourdough probiotic lactic acid bacteria proteolytic system of gluten and their role in the degradation of proline‐rich α‐gliadin peptides with enhanced organoleptic features to ensure quality and safe food for CD patients. It also holds promise to evolve a low cost, nutrient efficient and management in patients affected by celiac disease.

## Supplementary information

**Additional file 1: Figure S1. a** Mass spectrum of 33-mer degradation by GS 33, **b** Mass spectrum of 33-mer degradation by GS 143, **c** Mass spectrum of 33-mer degradation by GS 181, **d** Mass spectrum of 33-mer degradation by GS 188. **Figure S2. a** 33-mer peptide as control. **b** 33-mer peptide degraded by consortium of GS 33, GS 143, GS 181 and GS 188 after 6 h incubation. **Figure S3**. **a** Chromatogram showing degradation of 33-mer peptide by GS 33, **b** Chromatogram showing degradation of 33-mer peptide by GS 143, **c** Chromatogram showing degradation of 33-mer peptide by GS 181, **d** Chromatogram showing degradation of 33-mer peptide by GS 188. **Figure S4. a** Precursor peptide match 1 for 33 mer fragment degraded by the consortium. Peptide sequence: (Q)LPYPQPQPF(-);1086.5521 (m/z value); score: 9.1588; b & y ion cut pattern: b2b4b5b5*b6b7b8b9*y2y3y4y5y6y7y8y8*y9, **b** Precursor peptide match 2 for 33 mer fragment degraded by the consortium. Peptide sequence: (Q)PQLPYPQPQLPYPQPQLPYPQPQPF(-);1061.8385 (m/z value); score: 8.8206; b & y ion cut pattern: b2b2*b3b3*b4b4*b7b7*b8b9b9*b10b10*b11b12*b17b17*b21b22*b23y1y2y3y4y4*y5y5*y6y6*y8y9y9*y10y11y12y18y19*y20y25, C. Precursor peptide match 3 for 33 mer fragment degraded by the consortium. Peptide sequence: (Q)PQLPYPQPQLPYPQPQPF(-); 1067.9156 (m/z value); score: 8.8151; b & y ion cut pattern: b2b2*b3b3*b4b7b7*b9b9*b10b10*b11b12*b16y1y2y3y3*y4y4*y5y5*y6y6*y7y8y9y9*y10y11y12y12*y18, D. Precursor peptide match 4 for 33 mer fragment degraded by the consortium. Peptide sequence: (-)LQLQPFPQPQLPYPQPQLPY(P); 798.0159 (m/z value); score: 8.5843; b & y ion cut pattern: b2b2*b3b3*b4b6b8b8*b10b10*b11b12b17b18y2y3y5y6y7y9y9*y10y15y17*y20, E. Precursor peptide match 5 for 33 mer fragment degraded by the consortium. Peptide sequence: (-)LQLQPFPQPQL(P); 784.9256 (m/z value); Score: 9.2248; b & y ion cut pattern: b2b3b4b6b8b10b10*b11b11*y2y2*y3y4y5y7y8*y11y11*. **Figure S5.** Fragment cascade of 33-mer peptide degraded by consortium. Large bold arrow: cleavage between Q and L; large normal arrow: cleavage between Q and P; bold dotted arrow: cleavage between L and P; narrow dotted arrow: cleavage between Y and P. This schematic representation has been constructed based on the peptide matches obtained using ProteinLynx Global Server with high score (> 8) and also on b and y ion cut patterns of the peptides. **Figure S6. a** The amino nitrogen content of the sourdough samples. Bar graph was plotted incorporating sample mean (n = 3) and error bar (standard deviation) of individual isolate. * indicates that results were statistically significant at p < 0.05 when means of each treatment compared to the means of other treatment pair wise in Tukey’s HSD test in conjunction with ANOVA. **b** pH content of the sourdough samples. Line graph was plotted incorporating sample mean (n = 3) and error bar (standard deviation) of individual isolate. The results were statistically significant at p < 0.05 when means of each fermentation time compared in Tukey’s HSD test in conjunction with ANOVA. However, for neutral and CAD (Chemically Acidified Dough), no significant difference was observed at different time of fermentation.

## Data Availability

The data sets generated during and/or analysed during the current study are available from the corresponding author on reasonable request.
