# Supplementary material for: Gluten hydrolyzing activity of Bacillus spp isolated from sourdough
Source: Microb Cell Fact. 2020 Jun 12;19:130. doi: 10.1186/s12934-020-01388-z (PMC7291523; doi:10.1186/s12934-020-01388-z)
Supplement: Supplementary file 1 — Additional file 1: Figure S1. a Mass spectrum of 33-mer degradation by GS 33, b Mass spectrum of 33-mer degradation by GS 143, c Mass spectrum of 33-mer degradation by GS 181, d Mass spectrum of 33-mer degradation by GS 188. Figure S2. a 33-mer peptide as control. b 33-mer peptide degraded by consortium of GS 33, GS 143, GS 181 and GS 188 after 6 h incubation. Figure S3. a Chromatogram showing degradation of 33-mer peptide by GS 33, b Chromatogram showing degradation of 33-mer peptide by GS 143, c Chromatogram showing degradation of 33-mer peptide by GS 181, d Chromatogram showing degradation of 33-mer peptide by GS 188. Figure S4. a Precursor peptide match 1 for 33 mer fragment degraded by the consortium. Peptide sequence: (Q)LPYPQPQPF(-);1086.5521 (m/z value); score: 9.1588; b & y ion cut pattern: b2b4b5b5*b6b7b8b9*y2y3y4y5y6y7y8y8*y9, b Precursor peptide match 2 for 33 mer fragment degraded by the consortium. Peptide sequence: (Q)PQLPYPQPQLPYPQPQLPYPQPQPF(-);1061.8385 (m/z value); score: 8.8206; b & y ion cut pattern: b2b2*b3b3*b4b4*b7b7*b8b9b9*b10b10*b11b12*b17b17*b21b22*b23y1y2y3y4y4*y5y5*y6y6*y8y9y9*y10y11y12y18y19*y20y25, C. Precursor peptide match 3 for 33 mer fragment degraded by the consortium. Peptide sequence: (Q)PQLPYPQPQLPYPQPQPF(-); 1067.9156 (m/z value); score: 8.8151; b & y ion cut pattern: b2b2*b3b3*b4b7b7*b9b9*b10b10*b11b12*b16y1y2y3y3*y4y4*y5y5*y6y6*y7y8y9y9*y10y11y12y12*y18, D. Precursor peptide match 4 for 33 mer fragment degraded by the consortium. Peptide sequence: (-)LQLQPFPQPQLPYPQPQLPY(P); 798.0159 (m/z value); score: 8.5843; b & y ion cut pattern: b2b2*b3b3*b4b6b8b8*b10b10*b11b12b17b18y2y3y5y6y7y9y9*y10y15y17*y20, E. Precursor peptide match 5 for 33 mer fragment degraded by the consortium. Peptide sequence: (-)LQLQPFPQPQL(P); 784.9256 (m/z value); Score: 9.2248; b & y ion cut pattern: b2b3b4b6b8b10b10*b11b11*y2y2*y3y4y5y7y8*y11y11*. Figure S5. Fragment cascade of 33-mer peptide degraded by consortium. Large bold arrow: cleavage between Q [file 12934_2020_1388_MOESM1_ESM.docx]

**Figure S1 A Mass spectrum of 33 mer degradation by GS 33**

**Figure S1 B Mass spectrum of 33 mer degradation by GS 143**

**Figure S1 C Mass spectrum of 33 mer degradation by GS 181**

**Figure S1 D Mass spectrum of 33 mer degradation by GS 188**


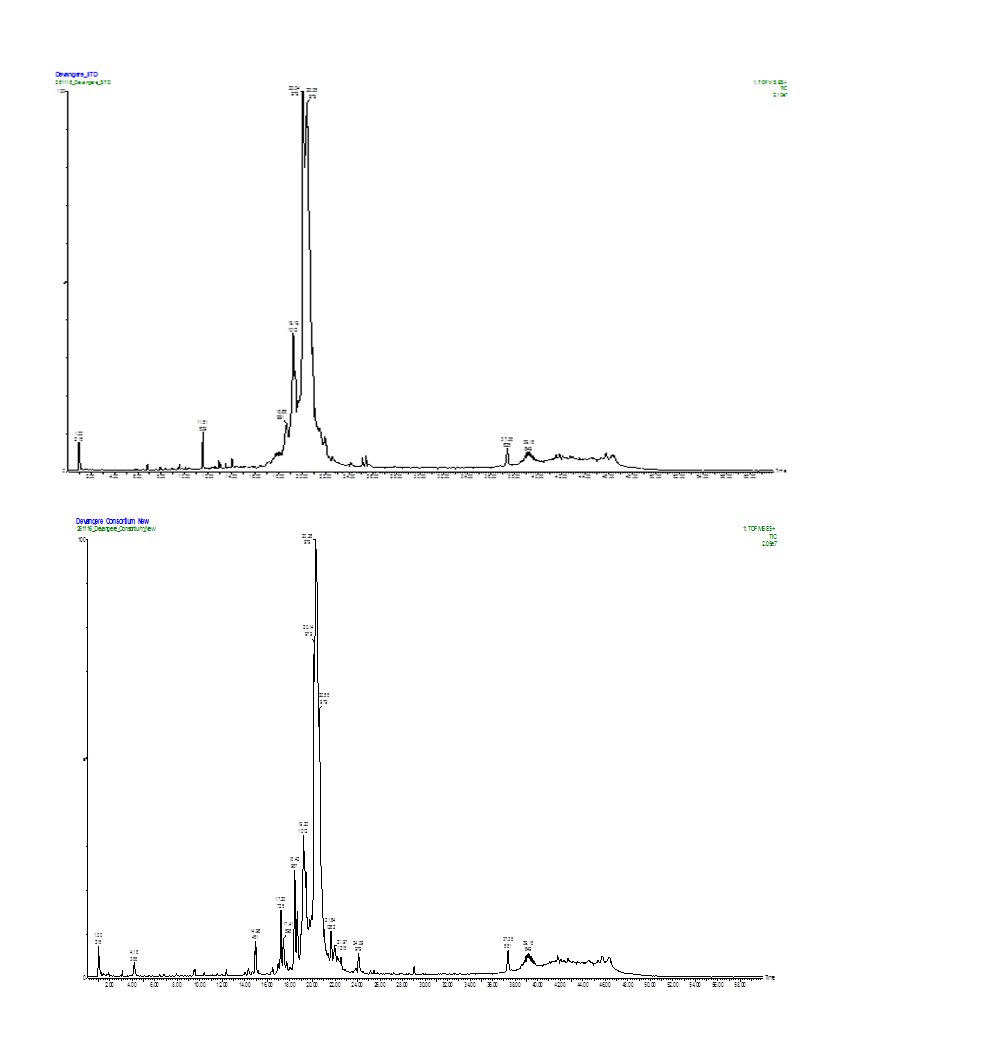


**Figure S2 A: 33 mer peptide as control. S2 B: 33 mer peptide degraded by consortium of GS 33, GS 143, GS 181 and GS 188 after 6 h incubation.**

**Figure S3 A Chromatogram showing degradation of 33 mer peptide by GS 33**

**Figure S3 B Chromatogram showing degradation of 33 mer peptide by GS 143**

**Figure S3 C Chromatogram showing degradation of 33 mer peptide by GS 181**

**Figure S3 D Chromatogram showing degradation of 33 mer peptide by GS 188**


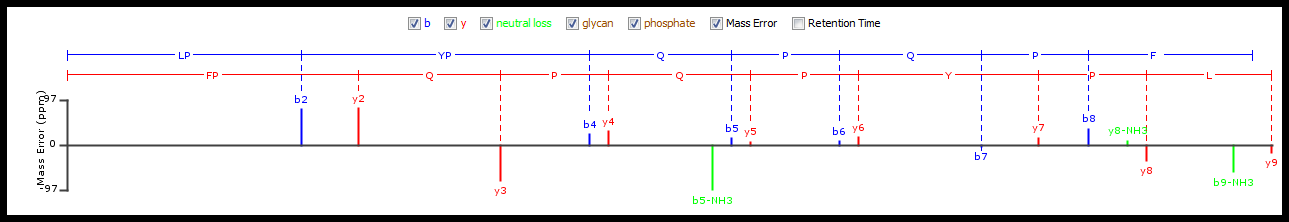


**Figure S4 A Precursor peptide match 1 for 33 mer fragment degraded by the consortium. Peptide sequence: (Q)LPYPQPQPF(-);1086.5521 (m/z value); score: 9.1588; b & y ion cut pattern: b2b4b5b5*b6b7b8b9*y2y3y4y5y6y7y8y8*y9**


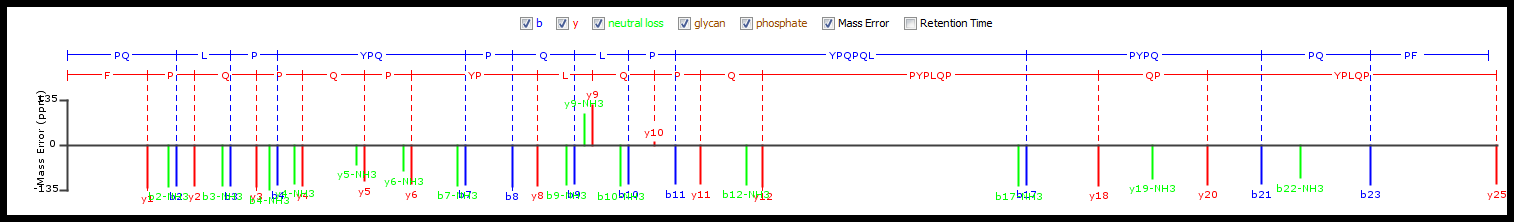


**Figure S4 B Precursor peptide match 2 for 33 mer fragment degraded by the consortium. Peptide sequence: (Q)PQLPYPQPQLPYPQPQLPYPQPQPF(-);1061.8385 (m/z value); score: 8.8206; b & y ion cut pattern: b2b2*b3b3*b4b4*b7b7*b8b9b9*b10b10*b11b12*b17b17*b21b22*b23y1y2y3y4y4*y5y5*y6y6*y8y9y9*y10y11y12y18y19*y20y25**


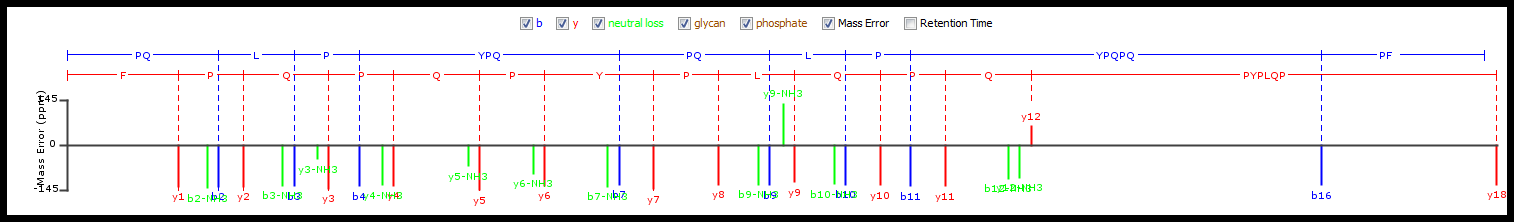


**Figure S4 C Precursor peptide match 3 for 33 mer fragment degraded by the consortium. Peptide sequence**: (Q)PQLPYPQPQLPYPQPQPF(-); 1067.9156 (m/z value); score: 8.8151; b & y ion cut pattern: b2b2*b3b3*b4b7b7*b9b9*b10b10*b11b12*b16y1y2y3y3*y4y4*y5y5*y6y6*y7y8y9y9*y10y11y12y12*y18


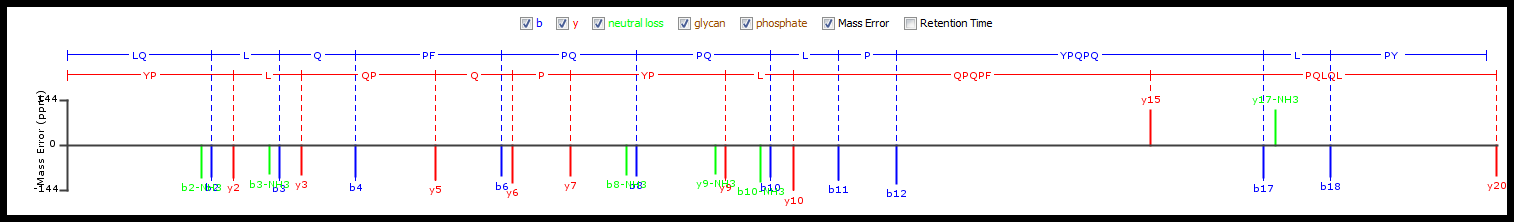


**Figure S4 D Precursor peptide match 4 for 33 mer fragment degraded by the consortium. Peptide sequence**: (-)LQLQPFPQPQLPYPQPQLPY(P); 798.0159 (m/z value); score: 8.5843; b & y ion cut pattern: b2b2*b3b3*b4b6b8b8*b10b10*b11b12b17b18y2y3y5y6y7y9y9*y10y15y17*y20


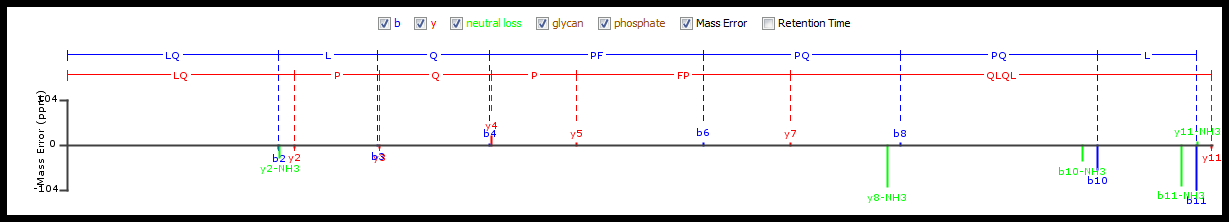


**Figure S4 E Precursor peptide match 5 for 33 mer fragment degraded by the consortium**. Peptide sequence: (-)LQLQPFPQPQL(P); 784.9256 (m/z value); Score: 9.2248; b & y ion cut pattern: b2b3b4b6b8b10b10*b11b11*y2y2*y3y4y5y7y8*y11y11*


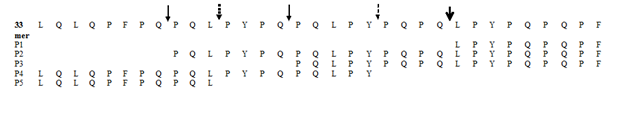
 **Figure S5: Fragment cascade of 33 mer peptide degraded by consortium. Large bold arrow: cleavage between Q and L; large normal arrow: cleavage between Q and P; bold dotted arrow: cleavage between L and P; narrow dotted arrow: cleavage between Y and P. This schematic representation has been constructed based on the peptide matches obtained using ProteinLynx Global Server with high score (>8) and also on b and y ion cut patterns of the peptides.**


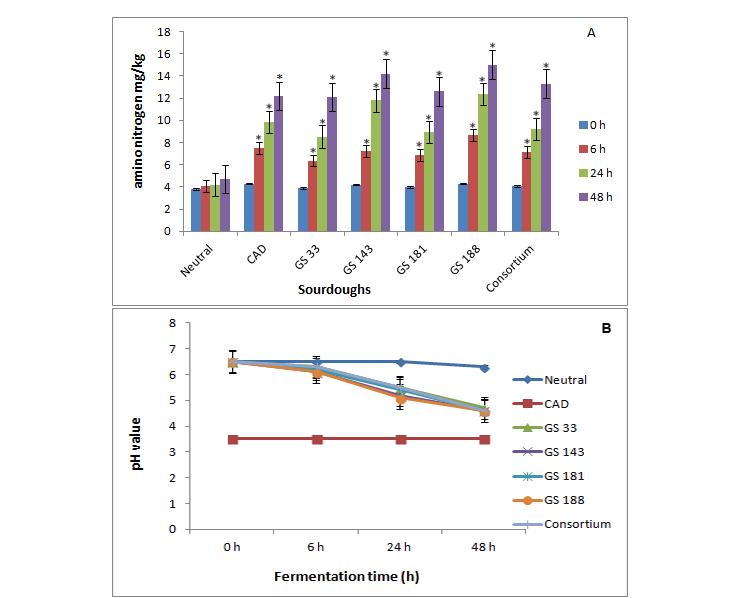


**Figure S6 A: The amino nitrogen content of the sourdough samples. Bar graph was plotted incorporating sample mean (n=3) and error bar (standard deviation) of individual isolate. * indicates that results were statistically significant at p<0.05 when means of each treatment compared to the means of other treatment pair wise in Tukey’s HSD test in conjunction with ANOVA. B: pH content of the sourdough samples. Line graph was plotted incorporating sample mean (n=3) and error bar (standard deviation) of individual isolate. The results were statistically significant at p<0.05 when means of each fermentation time compared in Tukey’s HSD test in conjunction with ANOVA. However, for neutral and CAD (Chemically Acidified Dough), no significant difference was observed at different time of fermentation.**
